# Effect of a simulation-based workshop on multidisplinary teamwork of newborn emergencies: an intervention study

**DOI:** 10.1186/s13104-015-1654-2

**Published:** 2015-11-12

**Authors:** Liisa Rovamo, Elisa Nurmi, Minna-Maria Mattila, Pertti Suominen, Minna Silvennoinen

**Affiliations:** HUCH Children and Adolescents, Helsinki University, BOX 94268, Helsinki, Finland; HUCH Department of Anaesthesiology and Intensive Care Medicine, Helsinki University, Helsinki, Finland; MinSim Oy, Lievestuore, Finland; Agora Center, University of Jyväskylä, Jyväskylä, Finland

**Keywords:** Newborn resuscitation, Simulation, Teamwork, Scoring

## Abstract

**Background:**

Video analyses of real-life newborn resuscitations have shown that Neonatal Resuscitation Program (NRP) guidelines are followed in fewer than 50 % of cases. Multidisciplinary simulation is used as a first-rate tool for the improvement of teamwork among health professionals. In the study we evaluated the impact of the crisis resource management (CRM) and anesthesia non-technical skills instruction on teamwork during simulated newborn emergencies.

**Methods:**

Ninety-nine participants of two delivery units (17 pediatricians, 16 anesthesiologists, 14 obstetricians, 31 midwives, and 21 neonatal nurses) were divided to an intervention group (I-group, 9 teams) and a control group (C-group, 6 teams). The I-group attended a CRM and ANTS instruction before the first scenario. After each scenario the I-group performed either self- or peer-assessment depending on whether they had acted or observed in the scenario. All the teams participated in two and observed another two scenarios. All the scenarios were video-recorded and scored by three experts with Team Emergency Assessment Measure (TEAM). SPSS software and nlme package were used for the statistical analyses.

**Results:**

The total TEAM scores of the first scenario between the I- and C-group did not differ from each other. Neither there was an increase in the TEAM scoring between the first and second scenario between the groups. The CRM instruction did not improve the I-group’s teamwork performance. Unfortunately the teams were not comparable because the teams had been allowed to self-select their members in the study design. The total TEAM scores varied a lot between the teams. Mixed-model linear regression revealed that the background of the team leader had an impact on differences of the total teamwork scores (D = 6.50, p = 0.039). When an anesthesia consultant was the team leader the mean teamwork improved by 6.41 points in comparison to specialists of other disciplines (p = 0.043).

**Conclusion:**

The instruction of non-technical skills before simulation training did not enhance the acquisition of teamwork skills of the intervention groups over the corresponding set of skills of the control groups. The teams led by an anesthesiologist scored the best. Experience of team leaders improved teamwork over the CRM instruction.

## Background

Neonatal resuscitation guidelines are published and updated regularly by the International Liaison Committee on Resuscitation (ILCOR) [1]. Unfortunately, video analyses of real-life newborn resuscitations have shown that Neonatal Resuscitation Program (NRP) guidelines are followed in fewer than 50 % of cases [2, 3]. More intensive teaching and training are therefore needed to improve the implementation of the resuscitation guidelines in order to improve the quality of neonatal resuscitation.

Simulation-based training is the standard method of teaching neonatal resuscitation [4]. Effective newborn resuscitation requires the integration of several technical skills, such as ventilation and intubation, in addition to non-technical skills such as behavioural/interactive (teamwork) and cognitive skills (knowledge and critical thinking) [5]. Although mask ventilation, intubation, and cardiac compression can be easily taught and rehearsed separately using high-fidelity newborn mannequins [6, 7], teaching co-ordinated teamwork to heterogeneous groups of medical specialists in an intensive emergency setting is a much greater challenge. The Joint Commission on Accreditation of Healthcare Organizations in the United States has recommended that team training should be taught to medical staff members to improve effective communication and cooperation during critical events [8]. A recent review article indicated that simulations, videos, and didactic lectures are effective methods of teaching teamwork [9].

Multidisciplinary simulation is used as a first-rate tool for the improvement of teamwork among health professionals [4, 9, 10]. Various specific team-rating scales have been developed to measure teamwork performances. These assessment tools are based on either self-ratings or observational team performance ratings in real and simulated settings [1, 11–14]. Commonly used assessment tools are the NRP Megacode Assessment form and also the team strategies and tools to enhance performance and patient safety (TeamSTEPPS) training, which have been used as a template to score neonatal resuscitation performance [1, 12]. The Team Emergency Assessment Measure (TEAM) is reported to be a valid and reliable instrument for rating teamwork during real adult medical emergencies [13, 14].

Approximately 70 % of medical errors (adverse events and ‘near misses’) have been reported to be due to human factors [15]. Most of these errors are caused by lapses in communication and safety culture of a hospital. Patients may be harmed as a result of these incidents. Crew resource management (CRM) and anesthesia non-technical skills (ANTS) are non-technical skills that can be defined as the cognitive and social human resources that complement technical skills. These non-technical skills contribute to safe and efficient task performance in teamwork [16]. Self-monitoring is a tool for acknowledging one’s own strengths and weaknesses but it has rarely been used in multidisciplinary simulation settings [17–19]. The evaluation of one’s own performance is essential for lifelong learning and it enhances error prevention in one’s work [17–21].

The principal aim of this study was to compare the TEAM scores in response to simulations between a control group (C-group) and an intervention group (I-group) that both participated in two subsequent scenarios of simulated newborn emergencies in real emergency rooms of two delivery units. The I-group teams received instruction on CRM and ANTS before the simulation and they also had instruction on conducting self- and peer-assessment relating to teamwork performance after the scenarios.

## Methods

Ninety-nine members of the medical staff of two large delivery hospitals in Helsinki volunteered for the present study between November 2012 and April 2013 (Table 1). Forty-four participants were from the Maternity Hospital of Helsinki University Hospital, which has around 6000 low-risk deliveries per year. Fifty-five came from Jorvi Hospital of Helsinki University Hospital, which has around 3800 low-risk deliveries per year.

**Table 1:** Demographic data of the participants of the simulation scenarios

	I-group (62 persons) (%)	C-group (37 persons) (%)
Multidisciplinary teams		
Physicians	31 (50.0 %)	16 (43.3 %)
Pediatricians	11	6
Registrar/Consultant	8*/3	1*/5
Anesthesiologists	10	6
Registrar/Consultant	6*/4	1*/5
Gynecologists	10	4
Registrar/Consultant	7/3	2/2
Midwives	17 (30.0 %)	14 (37.8 %)
Nurses	14 (20.0 %)	7 (18.9 %)
Main workplace		
Operating theatre	10 (16.1 %)	6 (16.2 %)
Delivery room	35 (56.5 %)	20 (54.1 %)
Neonatal intensive care unit	3 (4.8 %)	4 (10.8 %)
Maternity ward	13 (20.0 %)	7 (18.9 %)
Current working experience		
Less than 1 year	15 (24.2 %)*	2 (5.4 %)*
Between 1 and 5 years	18 (29.0 %)	13 (35.1 %)
Between 6 and 10 years	6 (9.7 %)	9 (24.3 %)
Between 11 and 15 years	8 (12.9 %)	5 (13.5 %)
Between 16 and 20 years	3 (4.8 %)	3 (8.1 %)
More than 20 years	12 (19.4 %)	5 (13.5 %)
Previous experience from neonatal resuscitation		
Ventilation	50 (80.6 %)	33 (89.2 %)
Chest compression	32 (51.6 %)	21 (56.8 %)
No previous experience of ventilation nor chest compression	12 (19.4 %)	4 (10.8 %)
Attended a lecture on neonatal resuscitation		
During past 12 months	29 (46.8 %)	11 (29.7 %)
During past 2 years	32 (51.6 %)	23 (62.2 %)
Never	1 (1.6 %)	3 (8.1 %)
Attended in practical training of neonatal resuscitation		
During past 12 months	31 (50.8 %)	12 (32.4 %)
During past 2 years	23 (37.7 %)	22 (59.5 %)
Never	7 (11.5 %)	3 (8.1 %)
Participated in a neonatal simulation session		
During past 12 months	22 (35.5 %)	9 (24.3 %)
During past 2 years	15 (24.2 %)	13 (35.2 %)
Never	25 (40.3 %)	15 (40.5 %)

* p < 0.05 Wilcoxon test

Approval from the Ethics Committee of Helsinki University Central Hospital was initially sought but the authors were informed that such approval was not actually required due to the observational nature of the study and lack of patient involvement. A signed consent was received from the participants to allow the use of the video-recordings from the simulation scenarios for research purposes.

**Fig. 1:**
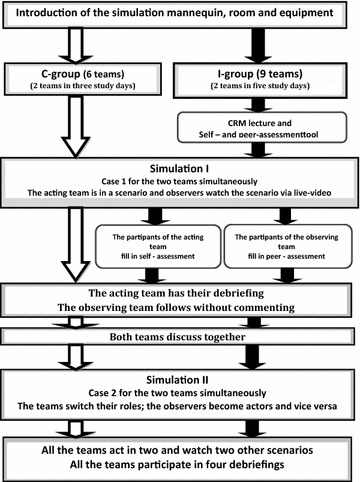
The study design

The participants registered for a single 1-day simulation training session from eight possible days provided. Participants created the teams themselves and the groups were drawn by lots to an intervention or a control group. Sixty-two professionals formed the I-group with nine teams, and 37 constituted the C-group with six teams (Fig. 1). All the teams had five to seven members with a composition that was similar to those teams in real emergency cases. Each team had a pediatric or an anesthesia consultant or a pediatric registrar who acted as a team leader. The team members included an obstetrician, a paediatrician, an anesthesiologist, a specialised neonatal nurse and one to two midwives (Table 1). Neonatologists were not available for the simulation scenarios. Each team participated in two scenarios and also observed two scenarios on a screen and was then debriefed after each scenario in both the I- and C-groups (Fig. 1).

The I-group attended an hour interactive lecture of the CRM and ANTS measures prior to running the first scenario. The CRM and ANTS measures comprise four skill categories (task management, team working, situation awareness, and decision making), which are in turn divided into 15 elements [16]. Self- and peer-assessment forms were used for the I-group to enhance the participants’ reflection of their own performance. The participants in the I-group performed either self- or peer-assessment during and immediately after each scenario depending on whether they had acted or observed.

The facilitators of the simulation sessions were a neonatologist and simulation instructor (L.R.), a specialist in simulation pedagogics (M.S.), and a specialised simulation nurse-instructor (M.M.M). The scenarios were designed as standard simulations of newborn emergencies. The scenarios were validated in preliminary simulation sessions for registrars. A case history was provided prior to the scenario. The first optional scenario was a sick newborn with breathing difficulty secondary to a serious infection or a newborn with asphyxia after birth. The second optional scenario was a newborn with hypovolemic shock due to either placental abruption or a navel cord interruption. All participants were given a similar introduction to the simulation mannequin, emergency room, and all the necessary equipment. Each scenario lasted 15–20 min and was followed by a 40 min debriefing session. At the beginning of the debriefing, each participant in the scenario gave their impression of their own performance. Both technical and non-technical issues related to performance and teamwork were discussed during the debriefing. Potentially serious clinical errors were pointed out either by the team members or the facilitators.

The simulation scenarios were run in the emergency rooms of the delivery units of the Maternity Hospital and Jorvi Hospital with the existing equipment (in situ training). A high-fidelity newborn mannequin was used in the simulation scenarios (Newborn HAL^®^ S3010; Gaumard, Miami, FL, USA). A recording and debriefing system (PRO+; Gaumard) was used for recording of all the scenarios. The live video stream of each scenario were captured and transmitted to the next room for the observers.

The teamwork performance of each team was scored by three anesthesiologists who independently viewed all the video-recordings. The first 10 min of video recording from a team’s performance in each scenario was used for the scoring based on the validation of scenarios. The raters were blinded to which teams were assigned to the intervention and control treatments. Teamwork performances were rated using the TEAM instrument with 11 items each with a score between 1 and 4 points [13]. The first two items dealt with leadership, the next seven items with teamwork, and the last two items with task management. The maximum TEAM score was 44 points. Additionally, an overall team performance rating was given to each team. The members of the team were expected to follow the Finnish national neonatal resuscitation guidelines [22].

SPSS software, version 17.0 (SPSS, Chicago, IL, USA) was used for the statistical analyses. Normally distributed demographic data of the participants and teamwork scores of the intervention and control teams were analyzed using a paired *t*-test. Cronbach’s alpha was used to test the internal consistency of the items on the forms for teamwork scoring. p-values of less than 0.05 were considered to indicate statistically significant differences.

The inter-rater reliability for the degree of agreement among the scores of the three coders (experts) for teamwork performance was tested with kappa variants. A kappa value was computed for all coder pairs and then the arithmetic mean of these estimates was used to provide an overall index of agreement. Conventionally, a kappa of <0.2 is considered poor agreement, 0.21–0.4 fair, 0.41–0.6 moderate, 0.61–0.8 strong and more than 0.8 near complete agreement [23].

The linear mixed-effects model regression analysis for repeated measures was used to define the predictors of teamwork performance. R package, version 3.0.3 (nlme package) was used in the analysis and values are reported as the values of L-R-ratio (D value).

## Results

### The comparison of the groups

The demographic data of the participants are presented in Table 1. The number of teams and participants were not equally divided between the study groups (Table 1). There were significantly more pediatric or anesthesia specialists in the C-group than in the I-group (p < 0.05). In the comparison of the groups there were significantly (p < 0.05) more participants in the I-group whose working experience was less than 1 year. The participants of the both groups had equal training of previous resuscitation and simulation practice. Forty percent of the participants in both teams participated in a simulation training for the first time and 16 percent had no previous experience of actual neonatal resuscitations (Table 1).

### Teamwork

**Fig. 2:**
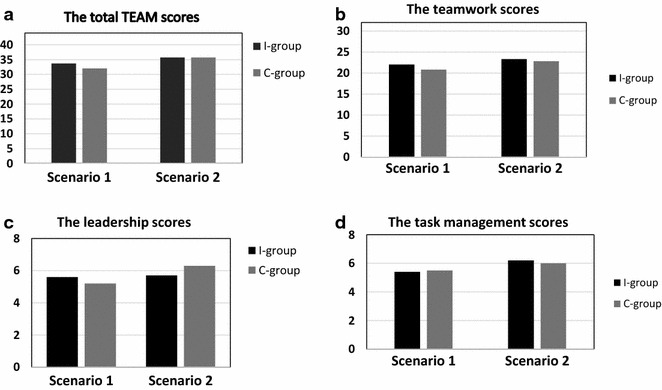
The comparison of the mean Team Emergency Assessment Measure scores. The total teamwork score (**a**), and its subgroups: leadership (**b**), teamwork (**c**) and task management scores (**d**) for two of the subsequent simulation scenarios for the intervention (*black bar*) and for control group (g*rey bar*)

The TEAM scores for leadership, teamwork, task management and total team scores for both groups are presented in Fig. 2. The total TEAM scores varied between 20.1 and 43.4 points in the scenarios. The mean leadership, teamwork, task management and total TEAM scores did not differ in the first simulation scenario between the I- and the C-group. There was no increase in the TEAM scoring between the first and second scenario between the groups.

Linear mixed-model regression analysis for the total teamwork scores and its subgroups was performed to predict the variation of the total teamwork scores in the repeated scenarios. The regression analysis revealed that the team leader accounted for consistent differences between the total teamwork scores (D = 6.50, p = 0.039). When an anesthesia consultant led a team the mean teamwork performance improved by 6.41 points over that of either pediatric consultant or registrar (p = 0.043). The teams of the larger delivery unit had slightly higher scores for leadership (D = 1.53, p = 0.017) and task management (D = 0.77, p = 0.048) than the teams from the smaller unit, but there was no significant difference in the total teamwork scores (p = 0.066). The instruction or repeated scenarios had no significant effect on the total teamwork scores neither the numbers of the participants or experiences of the midwives or neonatal nurses in the teams.

### TEAM scoring

The quality of the TEAM scoring instrument was evaluated in the neonatal emergency simulations. The TEAM scoring tool had good internal consistency, Cronbach’s alpha was 0.919 (p < 0.01) in simulated emergences. The overall index of agreement for the TEAM score between the three anesthesiologists was moderate 0.41. The inter-rater reliability varied between poor and moderate. (The coder 1 and 2; Cohen’s kappa weighted = 0.231, SE = 0.149; 95 % CI = −0.062 to 0.524; the coder 1 and 3 Cohen’s kappa weighted = 0.448, SE = 0.144; 95 % CI = 0.167 to 0.730 and the coder 2 and 3 Cohen’s kappa weighted = 0.540, SE = 0.111; 95 % CI = 0.321 to 0.758.)

## Discussion

The results of our study showed that the instruction of non-technical skills given immediately before the simulations were run did not increase scores for teamwork skills of the intervention groups over the skills of the control groups. However, the C-group tended to have more experience. The mean leadership, teamwork, task management and total TEAM scores did not differ in the first simulation scenario between the I- and the C-group. There was no increase in the TEAM scoring between the first and second scenario between the groups. A recent review by Weaver et al. [9] reported that simulation was involved in 68 % of the activities of healthcare teamwork training and was found to be a powerful learning method. Li et al. [24] showed significantly higher post-test scores in lecture and simulation groups when the didactic lecture was given before the simulated scenarios compared to simulation groups only. The present study did not support that the didactic lecture before simulation has increased teamwork skills. The positive predictor of teamwork in the teams was the team leader.

In their recent publication Halemek et al. attempted to recognize the specific roles of all members of the multidisplinary teams and evaluated their contributions to the overall teamwork, instead of evaluating the contribution made by single individuals such as the team leader [25]. The midwives’ role is to be as a first-line actor in unexpected emergencies of a delivery unit. However, in our present study the midwives or nurses had no discernable impact on the total teamwork scores. The larger of the two delivery hospitals had slightly higher teamwork scores in leadership and task management subgroups, which might have resulted from more practice in real life of leadership and task management.

The physicians have to work as team leaders because of the nature of their profession. In our study, when an anesthesiologist led a team, the mean teamwork scores improved significantly. There are no published evaluations of team leadership of anesthesiologists in studies as far as we are aware. A recent review by Siassakos et al. reported that the variation in team efficiency correlated positively with their teamwork performance but not with individual team members’ knowledge, skills or attitudes [26]. However, Siassakos et al. noted a wide variation in the performance and analysis of multicenter simulation records. ‘Safe’ teams tended to declare an emergency earlier, and hand over in a more structured way, and use closed-loop communication. The capability and experience of the team leader seemed to be more important than his/her seniority. Sakran et al. surveyed the prospective teamwork assessment of trauma teams and showed that the experience of the attending surgeon affected the clinical efficiency compared to those teams directed by less experienced surgeons, who also took significantly longer time to complete the survey [27]. The same authors also speculated that more formal leadership training could potentially improve patient care and should be included in surgical education. It seems therefore that in our institutions pediatric consultants and registrars in particular would require more training and practice to become a better team leader, and that the instruction of CRM was not enough. Many of our pediatricians and junior doctors worked in a general clinic during the day and they only encountered newborn emergencies when they were on call, whereas anesthesiologists are more often in charge when emergencies arise in operation suites. Yeung et al. surveyed the relationship between team leadership skills and quality of cardiopulmonary resuscitation in an adult cardiac-arrest simulation. There were an association between team leadership skills and cardiac-arrest simulation test score. Teams led by leaders with the best leadership skills performed higher quality cardiopulmonary resuscitation with better technical performance [28].

Our simulations were performed in situ and the personnel formed realistic clinical multidisciplinary teams. In recent perinatal study conducted by Riley et al. [29] a significant reduction of patient harm was found in an in situ simulation programme. Riley et al. also reported a significant and consistent improvement of 37 % in perinatal morbidity between the pre- and post-intervention periods for a hospital engaged in simulation programme training. Very recently Rubio-Gurung et al. [30] described a significant decrease in hazardous events in neonatal resuscitations after multicenter simulation programme training in situ. In our study, the training and elucidation of CRM principles for the I-group before the first scenario and the subsequent simulations in situ seemed to improve the quality of the teamwork of inexperienced professionals such as the pediatric registrar.

We used TEAM as an assessment instrument to evaluate the teamwork of newborn emergency teams in simulation training environments [13, 14]. Earlier studies of teamwork in newborn resuscitation usually used the NRP Megacode Assessment Form as a template for scoring NRP performance [1]. TEAM has also been used to evaluate the multidisciplinary teamwork of resuscitation teams, either simulated or in ‘real’ settings in cardiac and trauma resuscitation of adults [13, 14]. We tested the reliability of the teamwork assessment instrument in newborns. The teamwork scale showed good internal consistency. Moderate agreement between the raters’ scorings of teamwork was obtained. It seems therefore that our assessment instrument, TEAM, is also suitable for the evaluation of multidisciplinary teamwork for newborn emergencies.

The self- and peer-assessment of the simulation-based workshop in our study did not increase the total teamwork scores in the intervention groups. The evidence in the literature suggests that physicians have a limited ability to self-assess accurately. A number of studies have found the lowest accuracy in self-assessment among physicians was found among the least skilled and also among those who were the most confident [31].

## Limitation

This study had several limitations. First, the study was carried out in two different delivery hospitals and the teams were allowed to self-select their members. Therefore, the groups were not equally experienced in working years and or in composition: there were also differing numbers of midwives, paediatricians or anesthesiologists in each team. However, the specialties of the clinical staff of the teams could only be ascertained on arrival to the respective simulation session on each study day due to their availability from more pressing duties.

Another limitation in our study design was that we could not recruit similar numbers of teams or more teams for our group comparisons because of the limited number of hospital staff members.

The third limitation was that in our country it is not compulsory to attend to the neonatal life support courses every second year as in many European countries and USA. It is only the recommended to take a part in lectures and practical training of neonatal resuscitation. However, the groups did not differ in previous experience from neonatal resuscitation or attendance on a lecture on neonatal resuscitation and practical training.

The two sets of the scenarios themselves might not be totally comparable (Fig. 1). The second scenario in which each team acted would be expected to be more demanding in relation to teamwork and leadership; because in that particular scenario the newborn had breathing difficulties and he had also bled. The newborn needed the support of breathing and he also needed a blood transfusion at the same time, whereas in the first scenario the newborn had only breathing difficulties.

## Conclusion

Emergency situations occur in high-risk environments such as delivery units every week. It is known that 70 % of mistakes in medicine are due to human errors [8]. The main areas to improve are communication and safety culture. Team training has been recommended to improve communication between medical personnel during critical events. Multidisplinary team simulation training in situ allows participants to act in their own roles as in real emergencies. However, there is no consensus how to assess and teach teamwork and crisis management.

The aim of the present study was to compare the TEAM scores in simulations between a control group and an intervention group. The I-group attended a CRM and ANTS instruction before the first scenario. Our study demonstrated that the instruction of non-technical skills (CRM and ANTS) given immediately before the simulations did not increase scores for teamwork skills of the intervention groups over the skills of the control groups. However the C-group tended to have more experience.

In our study the team leader had an impact on differences of the total teamwork scores. When an anesthesiologist led a team, the mean teamwork scores improved significantly. Many of our pediatricians and junior doctors worked in a general clinic during the day and they only encountered newborn emergencies when they were on call, whereas anesthesiologists are more often in charge when emergencies arise in operation suites. Many of the pediatrics consultants and registrars, who are not working regularly in delivery units, might need more regular and individualized training in neonatal emergencies as a team leader to increase patients’ safety.

Future research should focus on exploring the transfer of clinical skills, teamwork, and self-assessment from the simulation environment to authentic environments. The effect of teamwork and leadership on a newborn’s outcome should then be evaluated in clinic trails on newborn emergencies.

## Abbreviations

IRR: the inter-rater reliability
ANTS: anesthesia non-technical skills
CRM: the crisis resource management
ILCOR: The International Liaison Committee on Resuscitation
NRP: Neonatal Resuscitation Program
TEAM: Team Emergency Assessment Measure
TeamSTEPPS: the team strategies and tools to enhance performance and patient safety
